# The impact of hepatitis viruses on chronic lymphoproliferative disorders; preliminary results

**Published:** 2010-08-25

**Authors:** AM Vladareanu, C Ciufu, AM Neagu, M Onisai, H Bumbea, C Ciufu, AM Vintilescu, C Dobrea, V Arama, R Mihailescu, S Arama

**Affiliations:** * Hematology Department, University Emergency Hospital BucharestRomania; ** ‘Victor Babes’ National Institute of Development and Research BucharestRomania; *** ‘Matei Bals’ Institute of Infectious Diseases BucharestRomania; **** Pathophysiology Department, ‘Carol Davila’ University of Medicine and Pharmacy BucharestRomania

## Abstract

The aim of this study is to analyze a group of patients with chronic lymphoproliferative disorders associated with B, C, D hepatitis viral infection. This group of chronic lymphoproliferative disordered patients with associated hepatitis viral infection has been diagnosed and monitored in the Hematology Department of the University Emergency Hospital of Bucharest, between December 2007 and January 2009. Our study is meant to observe the influence of the viral infection on clinical and biological evolution of the enrolled patients.

The diagnosis of the chronic lymphoproliferative disorder was based on the bone marrow / lymph node biopsy and flow–cytometry analysis. The positive diagnosis for hepatitis viral infection was established by ELISA serological tests and viremia was performed by TaqMan method at INBI ‘Matei Bals’ Bucharest.

The analyzed group is made up of 41 patients, 25/41 (60,97%) females and 16/41 (39,02%) males, with ages: 20–50 years old – 6/41 (14,63%), 51–70 years old – 23/41 (56,09%) and over 71 years old – 12/41 (29,26%) patients. The histological types of CLD: B–cell non–Hodgkin's lymphoma – in 28/41 (68,29%) patients, T–cell non–Hodgkin's lymphoma – 2/41 (4,87%) patients, Hodgkin's lymphoma – 2/41 (4,87%), chronic lymphocytic leukemia  – 7/41 (17,07%), Waldenström disease – 2/41 (4,87%) patients. Regarding the type of CLD, 19/41 (46,34%) of the patients have an aggressive type of CLD and 22/41 (53,65%) a non–aggressive type of CLD. The hepatitis viral infection distribution in our patients: 14/41 (34,14%) have HBV infection, 24/41 (58,53%) have HCV infection, double/triple association of viral infection was found in 3/41 (7,31%) patients. Within HBV infection subgroup 9/14 (64,28%) patients have an aggressive type of CLD and 5/14 (35,71%) patients have a non–aggressive type, whereas within the group with HCV infection we found a different distribution: 9/24 (37,5%) patients with aggressive type and 15/24 (62,5%) with non–aggressive type of CLD. The clinical parameters monitored were: B signs were present in 19/41 (43,34%) patients, the superficial or profound adenopathies –were found in 29/41 (70,73%) patients, hepatomegaly – in 38/41 (92,68%) patients, splenomegaly – in 21/41 (51,21%) patients,  extra–nodal involvements  in 10/41 (24,39%) patients and most frequent in the non–aggressive type of CLD – 6/10 (60%) patients. The hematological and biochemical parameters were: the presence of anemia and thrombocytopenia – found in a small number of patients; lymphocytosis – positive in 33/41 (80,48%) patients, most with HCV infection and non–aggressive type of disease, the presence of autoimmune hemolytic anemia – in 4/41 (9,75%) patients, cryoglobulins – 8/41 (19,51%) patients, all with HCV infection; also the liver function was monitored. Antiviral therapy was administered to 12/41 (29,26%) patients – Lamivudine to  8/41 (19,51%) patients and Ribavirine/Interferon to 4/41 (9,75%) patients. Chemotherapy was given in 32/41 (78%) patients. Monoclonal antibodies anti CD20 (Rituximab) therapy was associated in 6/41 (14,63%) patients.

**Conclusions.** A high incidence in female sex of over 50 years old was noticed. A strong association between B–cell chronic lymphoproliferative disorders and hepatitis viral infection B, C, D was revealed, the most frequent being the C hepatitis virus, associated with the non–aggressive type of CLD, extra–nodal involvement, splenomegaly, lymphocytosis, cryoglobulins, cytolysis and colestasis. The clinical and biological disease history will be monitored during chemotherapy and antiviral treatment.

## Background

### HBV infections and chronic lymphoproliferative disorders

The hepatitis viral infections are frequent in patients with chronic lymphoproliferative disorders, being involved in their pathogeny and contributing to the morbidity and mortality of these patients. HBV acute infection could evolve to spontaneous recovery in 90% of the cases or could become a chronic infection, the risk decreasing with age (5–10% in adults). The risk is higher in immunosupressed patients. Due to the fact that HBV has a special hepatic tropism, it was revealed that there are small quantities of non–replicative HBV–DNA or viral antigens in mononuclear cells from peripheral blood such as monocytes, B–lymphocytes, CD4 and CD8 T–lymphocytes, leading to the idea that the lymphocytes represent an extrahepatic viral reservoir.

### HCV infection and chronic lymphoproliferative disorders

The HCV acute infection is asymptomatic in 75% of the cases, becoming chronic in 80% of the cases, much more frequent than HBV infection, and evolving to hepatic cirrhosis in a period of 5–30 years. HCV is a hepatotrop virus, but it is able to infect and replicate in hematopoietic cells, a lot of studies showing the presence of RNA–HCV within B, T–lymphocytes and monocytes in patients with HCV infection. 

## Objectives

We have performed a retrospective and prospective study of patients with CLD associating hepatitis viral infections diagnosed in the Hematology Department of SUUB starting with December 2007. The objectives included the positive diagnosis of chronic lymphoproliferative disorder, the statistic analysis of clinical and biological parameters, the correlations between these parameters and the identification of prognostic markers.

The positive diagnosis was established according to the International Guidelines:

1. For chronic lymphocytic leukemia (CLL), after National Cancer Institute (NCI) (7) – lymphocytosis > 5 x 10^9^/L in peripheral blood and confirmation by flow–cytometry of lymphocytes [1].
2. For non–Hodgkin's lymphoma (NHL) – by assessing the malignant lymphoid population with special immunophenotype, by immunohystochemistry or immunophenotyping, using modified REAL [2] and WHO classifications [3, 4].

The inclusion criteria are: positive diagnosis of a B or T chronic lymphoproliferative disorder (established on leukocytosis with lymphocytosis, flow–cytometry on peripheral blood or bone marrow, bone marrow aspirate, bone marrow biopsy, lymph node biopsy immunohystochemistry) and infection with hepatitis viruses (B, C, D).

The exclusion criteria was the absence of hepatitis viruses infection.

## Material and methods

We monitored clinical parameters, obtained from patients' files, such as B signs (weight loss > 10% from body weight, fever, sweats), the presence of adenopathies, hepatomegaly, splenomegaly, the extra–nodal and bone marrow involvement, the clinical signs of liver disease (jaundice, peritoneal liquid, collateral circulation). The biological parameters monitored were:

- anemia – Hb < 11 g/dl
- thrombocytopenia PLT < 150.000/mm^3^
- lymphocytosis > 20% lymphocytes
- presence of autoimmune hemolytic anemia
- the value of serum LDH
- kidney tests (BUN, creatinine), uric acid
- liver tests (GOT, GPT, alkaline phosphatase, GGT), total bilirubine with fractions
- the presence of cryoglobulins
- coagulation changes

The blood cell count was performed in the Hematology Laboratory of The Hematology Department of University Emergency Hospital of Bucharest, including the morphologic analysis of the blood smear. The biochemical tests were performed in the Laboratory of SUUB.

## Results

The patients enrolled in this analysis were diagnosed and monitored in our department from December 2007 until January 2009.

In the group, there were 41 patients with CLD and B/C/D hepatitis viral infection, among whom 25 females (25/41=60,97%) and 16 males (16/41=39,02%). The median age of the group was of 64 (min 30, max 85) years old, and the patients' distribution according to the age was: 20–50 years old – 6/41 (14,63%) patients, 51–70 years old – 23/41 (56,09%) patients and over 70 years old – 12/41 (29,26%) patients. The epidemiologic investigation revealed that 4/41 (9,73%) patients had received blood transfusions before, 15/41 (36,58%) patients had surgery (apendicectomy, ovarectomy, gastrectomy, diagnosis or therapeutic splenectomy, laborious teeth surgery) and 4/41 (9,75%) patients associated both epidemiologic conditions for hepatitis viral infection.

Within the analyzed group, the predominant was the B–cell lineage – 39/41 (95,12%) patients, and, the T–cell lineage was found in only 2/41 (4,87%) patients.

**Figure 1:**
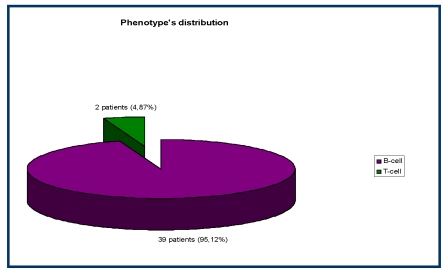
The B/T phenotype distribution of CLD in the study group

The patients' distribution according to the histological type of CLD is the following: diffuse large B–cell non–Hodgkin lymphoma – 13/41 (31,70%) patients, small B–cell NH lymphoma – 11/41 (26,82%) patients, follicular NH lymphoma – 3/41 (7,31%) patients lymphoblastic lymphoma – 1/41 (2,43%) patients, T–cell NH lymphoma – 2/41 (4,87%) patients, Hodgkin's lymphoma – 2/41 (4,87%) patients, chronic lymphocytic leukemia – 7/41 (17,07%) patients and Waldenstrom disease – 2/41 (4,87%) patients.

**Figure 2:**
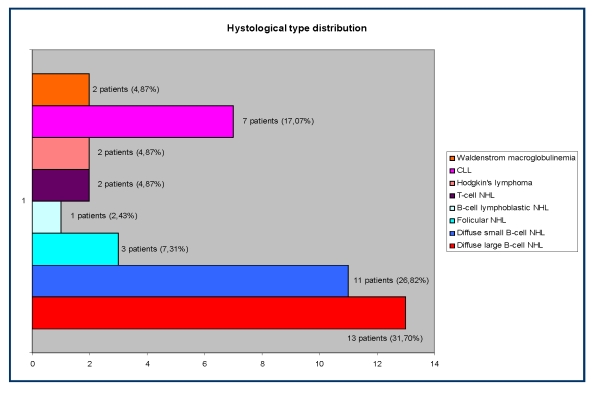
The distribution according to the histological type of the CLD in the study group

The aggressive form of CLD was found in 19/41 (46,34%) patients and the indolent type (non–aggressive) in 22/41 (63,5%) patients. 

The hepatitis viral infections associated in our group were: HBV infection in 14/41 (34,14%) patients, HCV infection in 24/41 (58,53%) patients, the double association HBV + HDV in 1/41 (2,43%) patients, HBV + HCV in 1/41 (2,43%) patients and the triple association HBV + HCV+ HDV also in 1/41 (2,43%) patients. 

**Figure 3:**
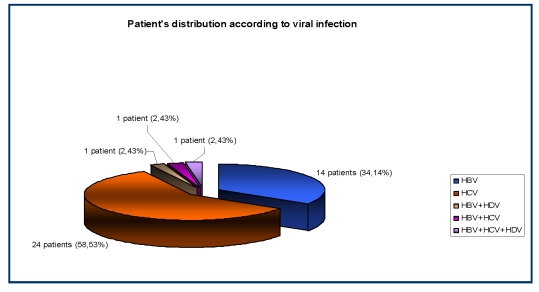
The distribution of patients according to the viral infection

Analyzing the correlation between the viral infection and the type of CLD (aggressive / indolent), we observed that within the group with HBV infected patients 9/14 (64,28%) have aggressive histological type and 5/14 (35,71%) patients have the indolent histological type of CLD, compared to the group with HCV infected patients in which 15/24 (62,5%) have non–aggressive histological type and 9/24 (37,5%) patients have an aggressive type of CLD.

**Figure 4:**
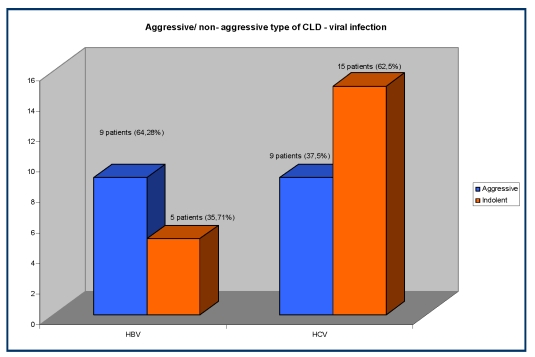
The correlation between the viral infection and the type of CLD

### Clinical signs

**Table 1:** Clinical signs

Clinical signs	
B signs	14/41 (43,34%)
Fever	1/19 (5,26%)
Weight loss	4/19 (21,05%)
Sweats	1/19 (5,26%)
All B signs	13/19 (68,42%)
Superficial/profound adenopathies	29/41 (70,73%)
Hepatomegaly	38/41 (92,68%)
Splenomegaly	21/41 (51,21%)
Extranodal involvement	10/41 (24,39%)
Jaundice	3/41 (7,31–)
Bleeding	2/41 (4,87%)
Peritoneal effusion	1/41 (2,43%)
Collateral circulation	3/41 (7,31%)

Clinical parameters were monitored. We concluded that the B signs were present in 19/41 (43,34%) patients: fever – 1/19 (5,26%), weight loss – 4/19 (21,05%), sweats – 1/19 (5,26%) and the association of all the three B signs – 13/19 (68,42%). 11/19 (57,89%) have an indolent histological type of CLD and 8/19 (42,10%) an aggressive type of CLD. 

The adenopathies (superficial/profound) were present in 29/41 (70,73%) patients; 9/29 (31,03%) associate HBV infection, 19/29 (65,51%) – HCV infection and 1/29 (3,44%) associate double infection HBV+HCV. 

Hepatomegaly was found in 38/41 (92,68%) patients, with equal distribution between the aggressive and the indolent CLD – 19/38 (50%) of patients. 

Splenomegaly was present in 21/41 (51,21%) patients. 5/21 (23,80%) with HBV infection, 13/21 (61,90%) patients – with HCV infection and 1/21 (4,76%) patients – with HBV+HCV/HBV+HDV/HBV+HD+HCV (one of each). 3/41 (7,31%) patients had splenectomy (diagnostic or therapeutic).

**Figure 5:**
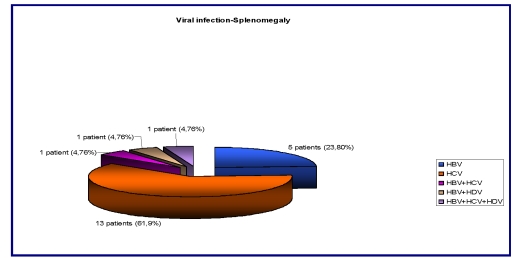
Correlation between viral infection and splenomegaly

The extra–nodal involvement was present in 10/41 (24,39%) patients; 4/41 (9,75%) patients with HBV infection and 6/41 (14,63%) patients with HCV infection. The clinical signs of hepatic failure were found in a small number of patients, such as: jaundice – 3/41 (7,31%), bleedings – 2/41 ( 4,87%), peritoneal effusion – 1/41 (2,43%), collateral circulation – 3/41 (7,31%). 

The hematological parameters: the median hemoglobin was 12 (min 8,5, max 17,4), normal ranges being 11–15 g/dl. In our group of patients – 9/41 (21,95%) had anemia (Hb < 11 g/dl), 4/9 (44,44%) patients had HBV infection and HCV infection, and 1/9 (11,11%) had triple viral association (HBV+HVC+HDV). We had lymphocytosis in 33/41 (80,48%) patients; 13/33 (39,39%) -  aggressive type of CLD: 4/13 (30,76%) patients were HBV infected, 8/13 (61,53%) were HCV infected and 1/13 (7,69%) patients were HBV+HCV infected; 20/33 (60,60%)– had the indolent type of CLD: 5/20 (25%) patients were HBV infected, 13/20 (65%) patients were HCV infected, 1/20 (5%) patients were HBV+HDV infected and 1/20 (5%) patients were HBV+HCV+HDV infected. 

**Figure 6:**
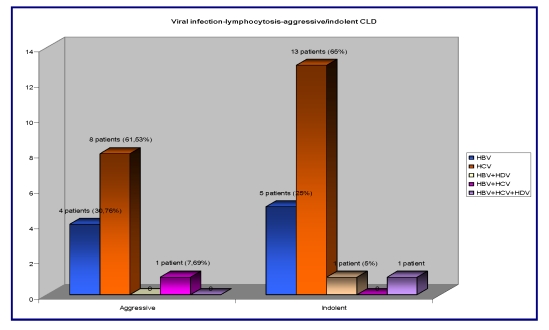
The correlation between the viral infection, lymphocytosis and aggressive/indolent type of CLD

Thrombocytopenia (ranges under 150.000/mm^3^) was revealed in 13/41(31,70%) patients. In this group, the patients had: 7/13 – had an aggressive type of CLD , from which 4/7 (57,14%) patients were HBV infected and 3/7 (42,85%) were HCV infected; 6/13 patients had an indolent type of CLD associated in 2/6 (33,33%) patients with HBV infection, 2/6 (33,33%) patients had HCV infection, 1/6 (16,66%) patients had double HBV+HDV infection and 1/6 (16,66%) patients had triple HBV+HCV+HDV infection.

**Figure 7:**
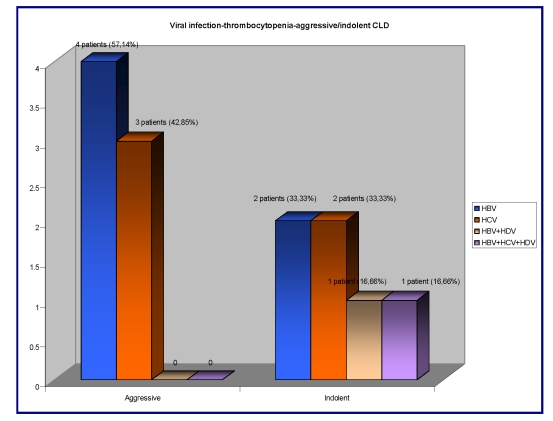
The correlation between the viral infection, thrombocytopenia and aggressive/indolent type of CLD

The bone marrow involvement was found in 23/41 (56,09%) patients.

The autoimmune features: autoimmune hemolytic anemia was present in 3/41 (7,13%) patients, all with HCV infection, 2 with indolent histological type of CLD and 1 patient with an aggressive type of CLD. The cryoglobulinemia was present in 8/41 (19,51%) patients, all with HCV infection, with equal distribution between the indolent and the aggressive type of CLD.  

### Biochemical features 

**Table 2:** Biochemical features

Biochemical features	
GOT (U/L)	61 (26–878)
GPT (U/L)	56 (25–1331)
AP (U/L)	100 (39–500)
GGT (U/L)	65 (22–1822)
TB (mg/dl)	0.9 (0.2–16.8)
LDH (U/L)	229 (129–1104)
The data is presented as a median value (minimum, maximum)	

High values of GOT (normal ranges 10–40 U/L) were revealed in 27/41 (65,85%) patients with: HBV infection 6/27 (22,22%), HCV infection 19/27 (70,37%), HBV+HDV 1/27 (3,70%) and HBV+ HCV 1/27 (3,70%) patients. In our group, GPT was over normal (normal ranges 10–37 U/L) found in 32/41 (78,04%) patients, out of whom 21/32 (65,62%) had HCV infection, 10/32 (31,25%) HBV infection and 1/32 (3,12%) patients double infection HBV+HCV. Only 8/41 (19,5%) patients had over normal serum alkaline phosphatase (normal ranges 40–150 U/L), out of whom 6/8 (75%) had HCV infection and only 2/8 (25%) had HBV infection. Monitoring the GGT levels, we observed high values (normal ranges 9–64 U/L) in 23/41 (56,09%) patients, most with HCV infection 14/23 (60,86%). The total bilirubine was high in 12/41 (29,2%) patients, most with HCV infection also 4/12 (58,33%) patients. 

The serum LDH, an important prognostic and activity marker of the hematological disease, was high in 17/41 (41,46%) patients, out of whom 9/17 patients  have an aggressive type of CLD associating the HBV infection in 3/9 (33,33%) patients, 5/9 (55,55%) patients had HCV infection and one of them associated a double infection HBV+HCV 1/9 (11,11%). The other 8 patients with elevated serum LDH have an indolent type of CLD:  3/8 (37.5%) patients with HBV infection and 5/8 (62,5%) patients with HCV infection.

**Figure 8:**
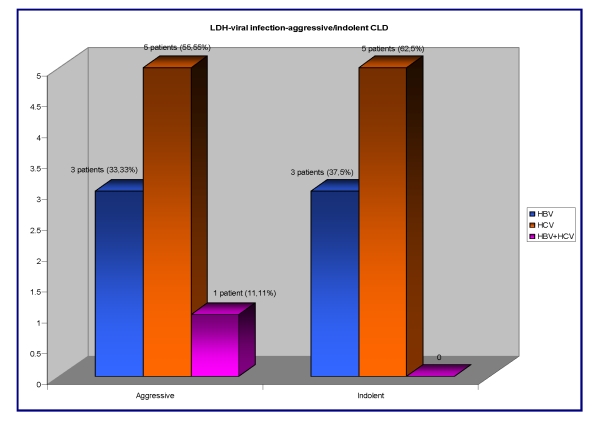
The correlation between the serum LDH, viral infection and aggressive/indolent type of CLD

## Therapy

The patients' therapy was also monitored. Thus, antiviral therapy was received by 12/41 (29,2%) patients: Lamivudine 8/41 (19,51%) patients, Ribavirine/Interferon 4/41 (9,75%) patients. Chemotherapy was administered to 32/41 (78,04%) patients: 5/41 (13,19%) had monotherapy (alkilant agents) and 27/41 (65,85%) patients received combined chemotherapy (CVP/CHOP/CHOEP +/– Rituximab, Fludarabine). We have to mention that 7/41 (17,07%) patients associated Rituximab – 2 patients with HCV infection and 5 with HBV infection. Chemotherapy associated to antiviral therapy was received by 9/41 (21,95%) patients, most of them with HBV infection 6/9 (66,66%).

## Discussions

Although HBV has a special hepatic tropism, it was revealed that there are small quantities of non–replicative HBV–DNA or viral antigens in mononuclear cells from peripheral blood such as monocytes, B–lymphocytes, CD4 and CD8 T–lymphocytes, leading to the idea that the lymphocytes represent an extrahepatic viral reservoir. The mechanism through which the viral genom persists and replicates within lymphocytes is yet not known. HBV could replicate in extrahepatic tissues like bone marrow and lymph nodes, being involved in extrahepatic pathology. An association of 30% between HBV and chronic lymphoproliferative disorders was demonstrated and cryoglobulins were found in 15% of the patients with HBV infection [5].

The reactivation of HBV infection could be the result of chemotherapy in patients with CLD associating HBV infection, but immunosuppression induced by CLD could be an independent risk factor for the reactivation [6]. The mechanism of viral reactivation represents the activation and increase of viral replication during immunosuppressive therapy, revealed by serum increase of DNA–HBV, the serum presence of AgHBe, followed by immune response mediated by T–lymphocytes, leading to an immune mechanism that destroys the infected hepatocytes. An analysis of the risk to develop a B–cell lymphoproliferative disorder in AgHBs positive patients showed that the risk of infection or viral reactivation is increased because of the direct immunosuppressive effect of the chronic lymphoproliferation and, also, HBV could have a part in lymphomagenesis through the persistence of small quantities of DNA–HBV in peripheral mononuclear cells, leading to a chronic stimulation of B–cells and clonal transformation in time [6]. 

As for the HCV, it was considered that the B–clone proliferation is happening in bone marrow and liver, as a response to chronic stimulation by HCV antigens such as E2 protein, this stimulated clone having a high risk of malignant transformation. The E2 protein has the binding site CD 81, representing the specific hepatocyte and B–lymphocyte receptor for HCV. The mechanism through which the HCV is leading to a chronic lymphoproliferative disorder is thought to be the chronic stimulation of B–lymphocytes through immune mechanisms [7].

Moreover, it was demonstrated that the HCV infected patients present the translocation involving the oncogene bcl–2 within their peripheral B–lymphocytes, representing a high risk of developing a non–Hodgkin lymphoma. 

A high prevalence of HCV antibodies and/or RNA–HCV in B–cell NHL patients was revealed and it was frequently associated with the mixed essential cryoglobulinemia. The exact relationship between HCV and cryoglobulinemia is not yet known, but it is certain that HCV is a chronic stimulus and that cryoglobulins are present in 50% of the HCV patients [8]. In HCV infection, we find autoimmune phenomena even in the absence of cryoglubulinemia and HCV could induce chronic lymphoproliferations [9]. The risk factors for cryoglobulinemia in HCV infected patients are: female sex, alcohol abuse, detectable RNA–HCV in serum, high gammaglobulins, high rheumatoid factor [5].

The association of HCV infection with T–cell non–Hodgkin's lymphomas and Hodgkin's lymphoma is not evident; only 5% of the patients were found with HCV infection and Hodgkin's disease [10].

Succesive studies made by Zignego et all in 1995 [11], 1996 [12] and 1997 analyzed HCV viremia in 35% patients with B–cell NHL, the most involved genotypes being 1b (61% of cases) and 2a (14% of cases). HCV was more frequent in low–grade malignity lymphomas. Other studies [13, 14, 15] do not reveal a higher prevalence of HCV infection in NHL patients, suggesting that HCV infection – NHL association is strictly related to geographic prevalence of HCV infection.

Most of the studies showed the relationship between HCV and low grade NHL, especially immunocytoma, but there is also a strong association with marginal lymphoma – Saadoun et al (2005) revealing a regression of a splenic lymphoma after antiviral therapy, thus suggesting the role of HCV in lymphomagenesis [16]. Published results showed the HCV prevalence in non-gastric MALT lymphomas – Arcaini et al (2007), the most prevalent sites being the skin, salivary glands and the socket [17]. The HCV role in gastric MALT lymphomas was demonstrated in 2002 by the isolation of RNA-HCV in gastric mucosa [18]. The most frequent extra–nodal sites of NHL associated to HCV infection are the liver and salivary glands [19, 20, 21, 22]. 

## Conclusions

The preliminary results of our study are in agreement with the literature data. A high frequency of chronic lymphoproliferative disorders associated with hepatitis virus infections was found in women, especially over 50 years old. B–cell CLD was the most frequent associated with hepatitis viral infection. In our group, the HCV infection is the most frequent hepatitis virus infection and highly associated with the non–aggressive histological type of CLD, the presence of adenopathies, hepatomegaly, splenomegaly and extra–nodal involvement. The lymphocytosis is significantly correlated with the HCV infection; the autoimmune phenomena were rare and found only in HCV infected patients. Moreover, the liver status was modified more, within the same subgroup of patients. 

All clinical, hematological and biochemical parameters will be analyzed in dynamics and correlated with the administered therapy and the dynamics of the disease course. 
